# *Neisseria gonorrhoeae* Sequence Type 16676 in Disseminated Infections, Minnesota, USA, 2025

**DOI:** 10.3201/eid3206.260126

**Published:** 2026-06

**Authors:** Daniel Evans, Allison LaPointe, Christine Peel, Khalid Bo-Subait, Elizabeth Dufort, Jenell Stewart, John Kaiyalethe, Bradley Craft, Matthew Plumb, Bonnie Weber, Laura Bohnker-Voels, Kelly Pung, Alyssa Mondelli, Jacob Garfin, Sarah Namugenyi, Paula Snippes-Vagnone, M. Elizabeth Gyllstrom, Kathryn Como-Sabetti, Ruth Lynfield

**Affiliations:** Health Protection Bureau, Minnesota Department of Health, St. Paul, Minnesota, USA (D. Evans, A. LaPointe, C. Peel, K. Bo-Subait, E. Dufort, J. Kaiyalethe, B. Craft, M. Plumb, B. Weber, L. Bohnker-Voels, K. Pung, A. Mondelli, J. Garfin, S. Namugenyi, P. Snippes-Vagnone, M.E. Gyllstrom, K. Como-Sabetti, R. Lynfield); University of Minnesota, Minneapolis, Minnesota, USA (J. Stewart); Hennepin Healthcare, Minneapolis (J. Stewart)

**Keywords:** bacteria, antimicrobial resistance, genomic surveillance, sexually transmitted infections, gonorrhea, Minnesota, United States

## Abstract

We summarize an outbreak investigation of *Neisseria gonorrhoeae* sequence type 16676 associated with disseminated gonococcal infections in Minnesota, USA, in 2025. This strain emerged rapidly, carried a plasmid with a tetracycline resistance gene, and encoded a *porB1a* allele. Prospective genomic surveillance enabled detection and epidemiologic investigation of this outbreak.

The sexually transmitted pathogen *Neisseria gonorrhoeae* can circulate from mucosal tissue at sites of exposure to other locations in the body, causing disseminated gonococcal infection (DGI) (*1*). In 2024, the Minnesota (USA) Department of Health initiated whole-genome sequencing (WGS) analysis of isolates from all DGI cases in the state (*2*). This article expands upon our previous study from 2024 (*2*).

In 2025, cases of DGI in Minnesota continued to occur at an elevated incidence rate compared with the 2020–2023 baseline. Minnesota state reporting rules require *N. gonorrhoeae* specimens from normally sterile sites to be submitted to the state public health laboratory. Analysis of those cases and linked specimens is considered enhanced surveillance and therefore deemed exempt from Institutional Review Board approval.

We performed WGS using the Illumina MiSeq, NextSeq, or MiSeq i100 platforms (https://www.illumina.com) and performed molecular epidemiologic analyses as previously described (*2*). Our genomic investigation showed that a new multilocus sequence type (ST), ST16676, emerged during the summer of 2025 (*3*,*4*). During June–September 2025, we sequenced 14 isolates from DGI cases whose genomes were assigned to ST16676. All 14 genomes encoded a *porB1a* allele, the tetracycline resistance gene *tet(M)*, the extended spectrum β-lactamase gene *bla*_TEM_, a Type XIV nonmosaic *penA* allele, and a gonococcal genetic island sequence (Figure 1; Appendix Table, Figure 1) (*3*–*6*). Those genomes did not match any documented *N. gonorrhoeae* sequence type by antimicrobial resistance profiles (*5*). Long-read sequencing (Oxford Nanopore Technologies, https://nanoporetech.com) of 4 ST16676 isolates consistently resolved the acquired *tet(M)* and *bla*_TEM_ genes on separate plasmid sequences of 42kb and 5.6kb and the *porB1a* allele on the bacterial chromosome.

**Figure 1:**
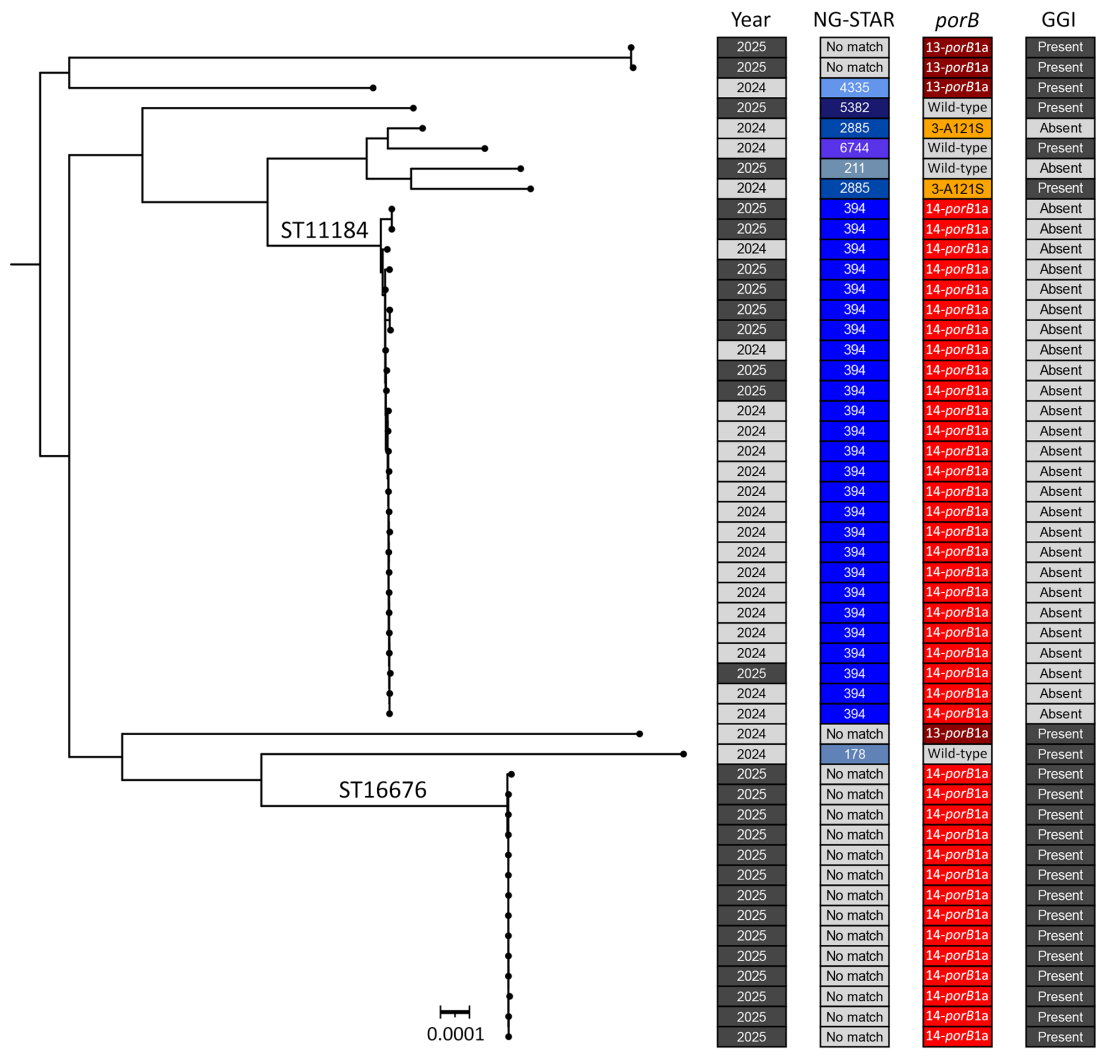
Midpoint-rooted phylogenetic tree constructed from an alignment of 1,626 core genes shared by 50 genomes of *Neisseria gonorrhoeae* isolates from study of outbreak of *N. gonorrhoeae* ST16676 among disseminated infections, Minnesota, USA, 2025. Annotations denote calendar year of specimen collection, *N. gonorrhoeae* sequence type by antimicrobial resistance (determined by using NG-STAR, https://ngstar.canada.ca), *porB* allele type, and presence of a GGI. Clades of genomes assigned to ST16676 and ST11184 are labeled on the tree. Scale bar represents substitutions per site. Figure was constructed using ITOL software (https://itol.embl.de). GGI, gonococcal genetic island; ST, sequence type.

In October 2025, we performed a global comparison of those genomes to others in the National Center for Biotechnology Information Pathogen Detection database (https://www.ncbi.nlm.nih.gov/pathogens). That comparison grouped the genomes into a cluster (PDS000214546.4) with 12 other genomes (Appendix Figure 2). Analysis of those 26 genomes using the Dryad version 3.0 pipeline showed that the Minnesota DGI genomes ranged in genetic similarity to each other by 0–62 (median 6) single-nucleotide polymorphisms (SNPs) and to the other 12 genomes by 215–320 (median 248) SNPs (Figure 2) (Dryad, https://github.com/wslh-bio/dryad). An iterative time-scaled phylodynamic analysis of those genomes showed that 28 of 32 iterations converged at early May 2025, weeks before the first case-patient sought care, as an estimated time of a most recent common ancestor for all 14 Minnesota genomes (Appendix) (*7*).

**Figure 2:**
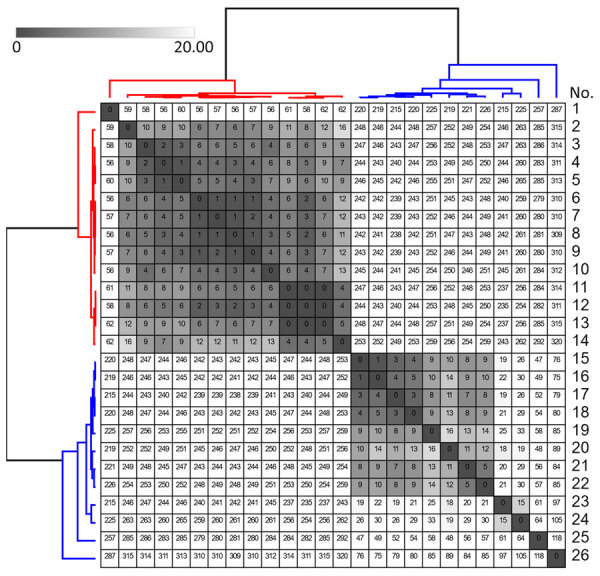
Reference-based pairwise single-nucleotide polymorphism (SNP) matrix of 26 *Neisseria gonorrhoeae* sequence type 16676 infections from study of outbreak of *N. gonorrhoeae* sequence type 16676 among disseminated infections, Minnesota, USA, 2025. Genomes numbered 1–14 (red) are from Minnesota disseminated gonococcal infection isolates in 2025. Genomes numbered 15–26 (blue) represent the other genomes grouped in the same National Center for Biotechnology Information Pathogen Detection cluster (PDS000214546.4.) in October 2025. SNP calls were clustered and displayed using Morpheus software (https://software.broadinstitute.org/morpheus). SNP calls <20 are highlighted in grayscale. Genome number 7 was used as an internal reference for calling SNPs with the Dryad version 3.0 pipeline (*7*).

Epidemiologists completed investigations of ST16676 DGI cases on the basis of findings from genomic surveillance. Of the 13 ST16676-infected case-patients interviewed, 12 (92.3%) resided within the Minneapolis-St. Paul-Bloomington metropolitan area; 11 (84.6%) were male and 2 (15.5%) female, and 9 (69.2%) were 15–44 years of age. Seven (53.8%) case-patients reported anonymous sexual encounters with multiple partners, 3 (23.1%) of whom reported substance use while doing so. Two (15.4%) reported having used doxycycline postexposure prophylaxis. Nine (69.2%) case-patients reported previous sexually transmitted infections; 4 (44.4%) reported gonorrhea and 5 (55.5%) reported HIV. Epidemiologic investigation confirmed a direct link between 2 cases whose isolates’ genomes were genetically identical at 0 SNPs. The 14th case-patient, who refused interviews, had an isolate that was identical at 0 SNPs to the 2 directly linked isolates, lived in an adjacent state, and received care for the infection in Minnesota.

Antimicrobial susceptibility test results were available in medical records of 10 (71.4%) of the 14 ST16676-infected patients. Consistent with results from *N. gonorrhoeae* sequence type by antimicrobial resistance and AMRFinderPlus analyses (*5*,*6*), all 10 isolates showed phenotypic resistance to tetracycline and ciprofloxacin and phenotypic susceptibility to ceftriaxone.

Prospective WGS detected the emergence of a tetracycline-resistant strain of *N. gonorrhoeae* that replaced the predominant DGI-associated strain from the previous year. The sudden emergence of a DGI-associated strain that carries both a *porB1a* allele (*8*) and a tetracycline resistance gene on a mobilizable plasmid poses epidemiologic concern, given the use of doxycycline postexposure prophylaxis to reduce potential illness and subsequent transmission risk of gonorrhea (*9*). In addition, the presence of 2 antimicrobial resistance genes on separately mobilizable plasmids highlights the importance of monitoring horizontal gene transfer in genomic surveillance of *N. gonorrhoeae*.

Our findings highlight the importance of DGI surveillance and the value of genomic surveillance for sexually transmitted infections. Prompt case investigations spurred by genomic analysis enabled epidemiologists to identify a direct link between DGI cases and notify a neighboring state health agency of transmission. Phylodynamic approaches also yielded insights into rates at which DGI-associated strains can emerge by estimating a timeline of weeks to months between the estimated time of a most recent common ancestor of a strain and the time at which infected case-patients sought care at healthcare facilities. Continuing prospective genomic surveillance, including performing large-scale studies of the evolution of DGI-causing *N. gonorrhoeae* strains, will help the field more thoroughly understand and intervene against this public health threat.

This article was preprinted at https://doi.org/10.64898/2026.01.09.26343522.

Appendix Additional information about outbreak of *Neisseria gonorrhoeae* sequence type 16676 in disseminated infections, Minnesota, USA, 2025
