# Supplementary material for: Neisseria gonorrhoeae Sequence Type 16676 in Disseminated Infections, Minnesota, USA, 2025
Source: Emerg Infect Dis. 2026 Jun;32(6):1022–5. doi: 10.3201/eid3206.260126 (PMC13245236; doi:10.3201/eid3206.260126)
Supplement: Appendix — Additional information about outbreak of Neisseria gonorrhoeae sequence type 16676 in disseminated infections, Minnesota, USA, 2025 [file 26-0126-Techapp-s1.pdf]

Article DOI: https://doi.org/10.3201/eid3206.260126

EID cannot ensure accessibility for supplementary materials supplied by authors. Readers who have difficulty accessing supplementary content should contact the authors for assistance.

Neisseria gonorrhoeae Sequence Type 16676 in Disseminated Infections, Minnesota, USA, 2025

Appendix

Appendix Methods

Single nucleotide polymorphism analysis of ST16676 genomes

Consistent with the standard Minnesota Department of Health bioinformatics protocol for genomic investigations healthcare-associated bacterial infections, we used the Dryad v3.0.0 pipeline to generate reference-based single nucleotide polymorphism (SNP) matrices of ST16676 genomes (*1*). Dryad v3.0.0 uses paired short read FASTQ files as inputs, performs quality control of sequencing reads using FastQC v0.11.8 (*2*), matches reads to microbial taxonomy using Kraken2 v2.0.8 (*3*), assembles genomes using Shovill v1.1.0 (*4*), assesses assembly quality using QUAST v5.0.2 (*5*), and calls SNPs among genome assemblies based on a provided reference genome using CFSAN SNP Pipeline v2.0.2 (*6*). The genome from the earliest collected ST16676 outbreak isolate from Minnesota was used as a reference for the CFSAN SNP Pipeline step.

Phylogenetic and phylodynamic analysis of ST16676 genomes

We annotated genome assemblies, constructed core genome alignments, and built phylogenetic trees of all *N. gonorrhoeae* genomes isolated from Minnesota DGI cases and of ST16676 genomes from Minnesota and NCBI using Bakta v1.9.4, Panaroo v1.5.0, and IQTree2 v2.3.6, respectively (*7*–*9*).

We performed 32 iterations of the TreeTime v0.11.4 algorithm with the midpoint-rooted tree, core genome alignment, and documented specimen collection dates of the Minnesota and non-Minnesota genomes assigned to the NCBI Pathogen Detection cluster PDS000214546.4 as of 31 October 2025 (*10*). These iterations covered all permutations of five binary parameters: whether (1) a clock rate was calculated by default settings or assigned by a pre-calculation step ("treetime clock" command, "–clock-rate" and "–clock-std-dev" flags), (2) the input tree for the iteration was the one generated by the Dryad pipeline or the version of it optimized when pre-calculating clock rates, (3) TreeTime was permitted to reroot the input tree during the iteration ("–keep-root" flag), (4) divergence times were calculated using jointly or marginally most likely inference settings ("–time-marginal" flag), and (4) polytomies were resolved greedily or stochastically ("–stochastic-resolve" flag). All 32 iterations allowed for phylogenetic covariance when estimating TMRCAs ("–covariation" flag), calculated 90% confidence intervals of mugration inferences ("–confidence" flag), and reported correlation coefficients of linear molecular clock models. We assessed the convergence of estimated TMRCAs in phylodynamic trees by comparing date ranges of 90% confidence intervals for each TreeTime iteration. Specimen collection dates were input as precisely as available to authors; all Minnesota ST16676 genomes were input to the day, whereas contextual genomes downloaded from NCBI were input to the month.

References

1. Wisconsin State Laboratory of Hygiene. Dryad [cited 2026 Mar 31]. https://github.com/wslh-bio/dryad

2. Andrews S. FastQC: a quality control tool for high throughput sequence data [cited 2025 May 27]. https://github.com/s-andrews/FastQC

3. Wood DE, Lu J, Langmead B. Improved metagenomic analysis with Kraken 2. Genome Biol. 2019;20:257. PubMed https://doi.org/10.1186/s13059-019-1891-0

4. Seemann T. Shovill: faster SPAdes assembly of illumina reads. 2017 [cited 2025 May 27]. https://github.com/tseemann/shovill

5. Mikheenko A, Prjibelski A, Saveliev V, Antipov D, Gurevich A. Versatile genome assembly evaluation with QUAST-LG. Bioinformatics. 2018;34:i142–50. PubMed https://doi.org/10.1093/bioinformatics/bty266

6. Davis S, Pettengill JB, Luo Y, Payne J, Shpuntoff A, Rand H, et al. CFSAN SNP Pipeline: an automated method for constructing SNP matrices from next-generation sequence data. PeerJ Comput Sci. 2015;1:e20. https://doi.org/10.7717/peerj-cs.20

7. Tonkin-Hill G, MacAlasdair N, Ruis C, Weimann A, Horesh G, Lees JA, et al. Producing polished prokaryotic pangenomes with the Panaroo pipeline. Genome Biol. 2020;21:180. PubMed https://doi.org/10.1186/s13059-020-02090-4

8. Tonkin-Hill G, MacAlasdair N, Ruis C, Weimann A, Horesh G, Lees JA, et al. Producing polished prokaryotic pangenomes with the Panaroo pipeline. Genome Biol. 2020;21:180. PubMed https://doi.org/10.1186/s13059-020-02090-4

9. Minh BQ, Schmidt HA, Chernomor O, Schrempf D, Woodhams MD, von Haeseler A, et al. IQ-TREE 2: new models and efficient methods for phylogenetic interence in the genomic era. Mol Biol Evol. 2020;37:1530–4. PubMed https://doi.org/10.1093/molbev/msaa015

10. Sagulenko P, Puller V, Neher RA. TreeTime: Maximum-likelihood phylodynamic analysis. Virus Evol. 2018;4:vex042. PubMed https://doi.org/10.1093/ve/vex042

Appendix Table. Summary of sequence typing by antimicrobial resistance (NG-STAR) alleles carried by the ST16676 strain

**NG-STAR AMR Allele**	**NG-STAR Allele Number**	**Allele Classification**	**Notable Allele Markers**
penA	14.001	Type XIV Non-mosaic	A517G
mtrR	512		A39T, G45D*
porB	14	*porB1a*	
ponA	100	Wild type	L421P*
gyrA	7		S91F, D95A
parC	3		S87R
23S	100	Wild type	

*AMR-associated point mutations that AMRFinderPlus detected in the gene sequences housing those alleles but did not influence NG-STAR classifications.

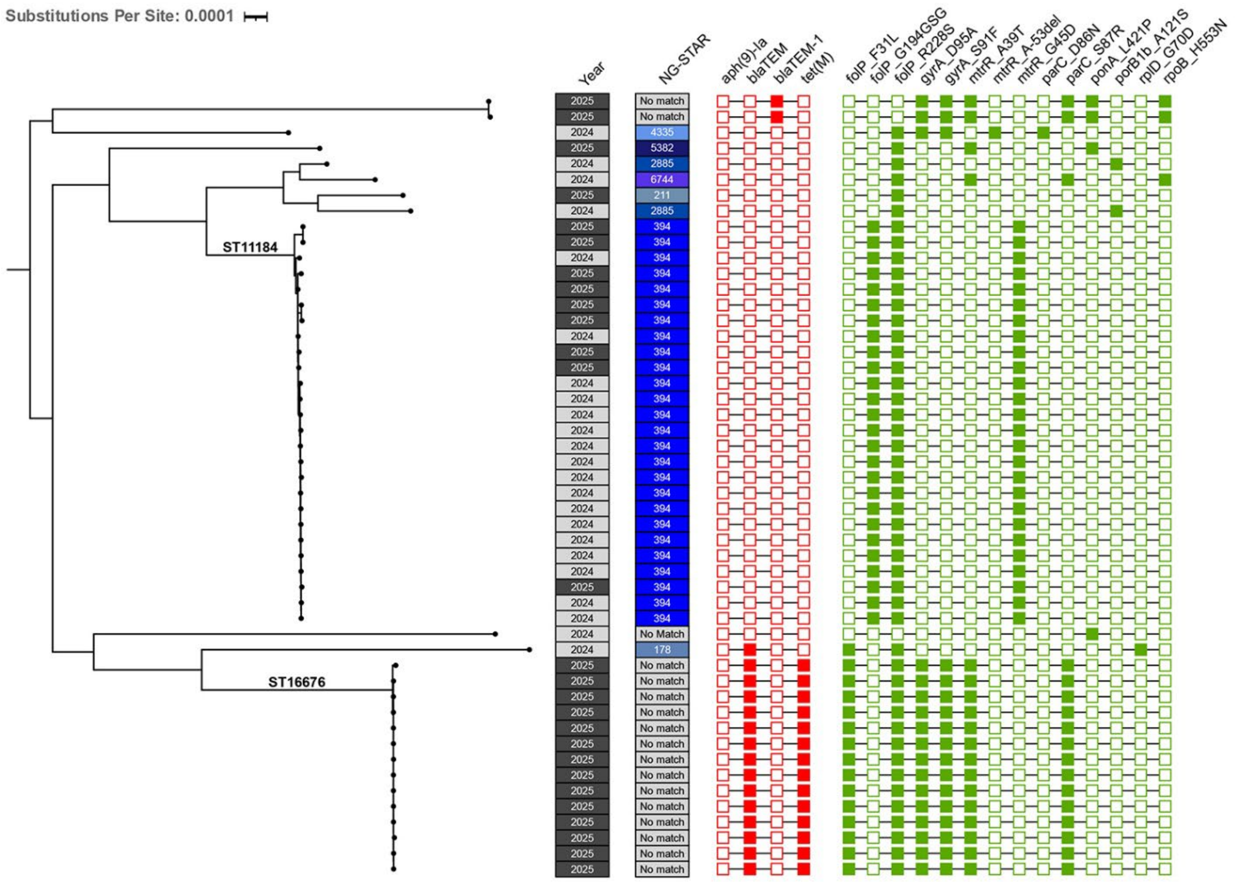

Appendix Figure 1. Midpoint-rooted phylogenetic tree constructed from an alignment of 1626 core genes shared by 50 genomes of *N. gonorrhoeae* isolates from disseminated infections in Minnesota in 2024 and 2025. Annotations denote calendar year of specimen collection, *N. gonorrhoeae* sequence type by antimicrobial resistance, and presence or antimicrobial resistance (AMR) markers detected by AMRFinderPlus. AMR markers shown in red denote acquired genes, and those shown in green denote point mutations. This figure was constructed using ITOL software (https://itol.embl.de/).

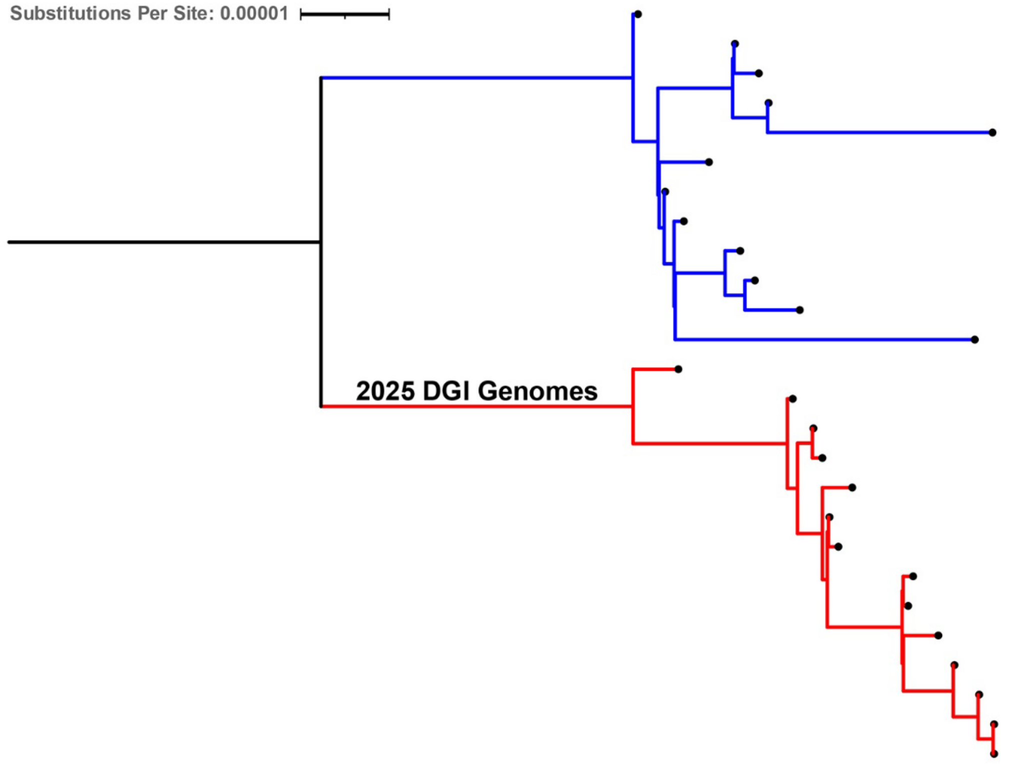

Appendix Figure 2. Midpoint-rooted phylogenetic tree constructed from an alignment of 2042 core genes shared among ST16676 genomes in NCBI Pathogen Detection cluster PDS000214546.4. Genomes from Minnesota DGI isolates in 2025 are highlighted in red, and those from other genomes in the cluster are highlighted in blue. This figure was constructed using ITOL software (https://itol.embl.de/).
